# Antimicrobial Utilization, Adverse Drug Reactions, and Associated Cost of Care in a Tertiary Care Hospital in Eastern India

**DOI:** 10.7759/cureus.76062

**Published:** 2024-12-20

**Authors:** Prasenjit Sutradhar, Mangala C Das, Sarada P Suna, Chaitali Pattanayak, Prem S Panda, Ipsita Debata

**Affiliations:** 1 Pharmacology, Kalinga Institute of Medical Sciences, Bhubaneswar, IND; 2 Medicine, Kalinga Institute of Medical Sciences, Bhubaneswar, IND; 3 Pharmacology and Therapeutics, Kalinga Institute of Medical Sciences, Bhubaneswar, IND; 4 Community Medicine, Kalinga Institute of Medical Sciences, Bhubaneswar, IND

**Keywords:** adverse drug reaction, antimicrobial resistance, antimicrobial use, cost of treatment, who aware index

## Abstract

Background

Antimicrobials considerably reduce infectious conditions, but their overuse and misuse contribute to various adverse drug reactions (ADRs) and antimicrobial resistance. In 2019, India adopted a national program to reduce antibiotic resistance for 2019-2021. Assessing antibiotic consumption among the out-patient and in-patient departments is paramount because it is the foundation for implementing and assessing antibiotic stewardship initiatives. This study aims to evaluate the usage pattern, ADR of antimicrobials and cost of treatment due to the use of antibiotics in patients admitted to selected departments of a tertiary care hospital in Eastern India.

Material and methods

An observational cross-sectional study was conducted on adult in-patients getting admitted to selected departments and ICUs of Kalinga Institute of Medical Sciences (KIMS), Bhubaneswar. Data were collected using a convenience sampling method from 417 patients during July 2022 to January 2024. Study tools included the Case Record Form, WHO prescribing Indicators, and WHO Causality Assessment scale. Descriptive statistics were presented using frequencies and percentages. The chi-square test was done to analyze the associations among categorical variables.

Results

Middle-aged individuals had a higher likelihood of hospital admission and antibiotic administration. The gender distribution was almost equal in the study population. It was found that ceftriaxone was the most common antimicrobial used in Medicine 56 (18.5%) and Surgery 49 (18.2%) departments. In Orthopaedics and Obstetrics and Gynaecology (O&G) departments, the most common antimicrobials used were cefuroxime 37 (20.3%) and cefixime 32 (22.2%), respectively. Piperacillin-tazobactam 59 (16.3%) was the most frequently utilized antimicrobial in the ICU. The majority of the antibiotics 800 (65%) were prescribed from the Watch group of the WHO AWaRe Index. The cost of antimicrobial treatment was highest in the ICU and the least in the O&G department. None of the participants had any serious adverse effects related to antimicrobials.

Conclusion

The most common group of antimicrobials used in this study were beta-lactams. Ceftriaxone, cefuroxime, and piperacillin-tazobactam were the most common drugs prescribed. It was found that this study does not correspond with the optimal values of WHO core drug use prescribing indicators. None of the participants had any serious adverse effects related to antimicrobials.

## Introduction

Antimicrobials have curtailed the global infectious diseases mortality rate [1]. These drugs are highly proficient in the management of a plethora of infections. Nevertheless, their harmful effects cannot be overlooked. While most antimicrobials considerably reduce infectious conditions, their overuse and misuse contribute to various adverse drug reactions (ADRs) and antimicrobial resistance [2].

Antimicrobial resistance (AMR) is a colossal burden today [2]. The World Health Organization (WHO) has perceived AMR as the biggest public health menace that humanity is encountering [1, 2]. In 2015, the World Health Assembly (WHA) ratified a global action plan (GAP) to fight AMR. These include reinforced information regarding antimicrobial resistance and strengthened infrastructure. It focuses on enhancing surveillance and research efforts, decreasing infections, maximizing the efficiency of antimicrobials, and ensuring a long-term commitment to combating AMR. The activities entailed in this aspect are ensuring sufficient water supply, sanitation, and hygiene, implementing measures to prevent and control infections, administering immunizations, providing laboratory services, educating staff, and managing the antibiotics supply chain [3].

Recently, there has been a heightened focus on using antibiotics to ensure quality and cost control. The Joint Commission for Accreditation of Hospitals compels healthcare institutions to conduct periodic assessments of antibiotic utilization. That includes assessing the prescribed types and reviewing the appropriateness of the chosen antibiotic and dosage [1, 2]. Several articles on antibiotic usage have depicted a high prevalence of irrational use of antimicrobial agents [3-8]. As per recent studies, prescription auditing necessitates the reviewer's conclusion to explain the rationale behind the antibiotic prescription [6, 9,10].

After considerable effort, the overall usage of antibiotics across India for systemic purposes decreased by about 30% from 2015 to 2017. In 2019, India adopted a national program to reduce antibiotic resistance for 2019-2021. It outlines the goals, actions, and protocols to be implemented to prevent the growing trend of antibiotic resistance. This includes promoting research on various areas of AMR at both the domestic and global levels [7]. Assessing antibiotic consumption among the out-patient and in-patient departments is paramount because it is the foundation for implementing and assessing antibiotic stewardship initiatives.

There is a dearth of literature regarding the evaluation of drug utilization patterns and adverse effects of antimicrobials in our institution. Hence, the present study was conducted. It was found that this study does not correspond with the optimal values of WHO core drug use prescribing indicators. It is anticipated that this study will contribute to the development of a more effective antimicrobial stewardship program. This study aims to evaluate the usage pattern, ADR of antimicrobials, and cost of treatment due to the use of antibiotics in patients admitted to selected departments of a tertiary care hospital in eastern India.

## Materials and methods

Study settings

An observational cross-sectional study was conducted among adult inpatients admitted to four departments, namely, Medicine, Orthopedics, Obstetrics & Gynecology, and Surgery, of Kalinga Institute of Medical Sciences (KIMS), Bhubaneswar, Odisha. The concerned wards and ICUs were routinely taken into consideration for this study. The study duration was 18 months, i.e., from July 2022 to January 2024. Data were collected using the convenience sampling method from 417 patients regarding their drug utilization and adverse drug reactions. Adult male and female patients aged 18-65 years admitted to the above departments and on one or more antimicrobials for at least 24 hours were included in the study. Patients who were unable to give information or incomplete information on patient bed head tickets or who did not give consent were excluded from the study. Approval from the Institutional Ethics Committee of KIMS was obtained on 18/7/2022 (Letter No. KIIT/KIMS/IEC/945/2022).

Data collection tool

The patients received the patient information sheet and were explained the intent of this research. They gave their consent before the study commenced. They had the right to withdraw their consent any time during the study without mentioning their reason. Data were collected on the 1st day and the 4th day of admission and on discharge or death of the participants.

After necessary consent and approval, demographic, laboratory parameters, and treatment details were ascertained from the bed head ticket and confirmed by interacting with the patients. All the information was gathered in a predefined case record form (CRF). The pattern of drug utilization was recorded, e.g., the number of antimicrobials in a prescription, the proportion of generic drugs prescribed, the percentage of encounters with an antibiotic, the proportion of injectables, the proportion of drugs enlisted in Essential Drug List (EDL), treatment cost, and hospital stay. Casualty assessment as per the World Health Organization-Uppsala Monitoring Centre (WHO-UMC) scale was performed. Monitoring of antibiotic consumption has been done using the WHO AWaRe (Access, Watch, Reserve) Index. Study Tools included Case Record Form, WHO prescribing Indicators, WHO-UMC Causality Assessment scale and WHO-AWaRe Index.

WHO prescribing indicators

The below-mentioned indicators were assessed among all the study participants irrespective of the nature and severity of their illnesses. It weighed the prescribing pattern of the institution. It included (1) the number of medications per prescription: which is the ratio of the total number of prescribed drugs and the number of prescriptions assessed, (2) the proportion of generic drugs: which is the ratio of the total number of prescriptions containing generic drugs and number of prescriptions assessed, (3) proportion of antibiotic-containing prescription, (4) proportion of injectables in a prescription, and (5) the percentage of drugs enlisted in EDL [11].

WHO-UMC Causality Assessment Scale

The WHO-UMC system has been developed in consultation with the National Centres participating in the Program for International Drug Monitoring and is meant as a practical tool for the assessment of case reports. It is basically a combined assessment taking into account the clinical-pharmacological aspects of the case history and the quality of the documentation of the observation [12]. 

WHO-AWaRe Index

In the AWaRe tool, antibiotics are divided into three categories: access, watch, and reserve. Each category is based on its respective effect on AMR. The “Access” antibiotics are characterized by their narrow spectrum of activity, typically resulting in fewer side effects, a reduced likelihood of antimicrobial resistance selection, and lower costs. They are strongly recommended for empiric treatment of common infections and should be readily available. Conversely, “Watch” antibiotics carry a higher risk of promoting antimicrobial resistance and are primarily prescribed for patients with more severe conditions, predominantly within hospital settings. Vigilant monitoring of these antibiotics is vital to prevent their overuse. “Reserve” antibiotics, however, are considered the last resort and should be employed only when dealing with severe infections caused by multidrug-resistant pathogens. Their use should be reserved for critical situations. The AWaRe classification underscores the importance of restricting the use of “Watch” and “Reserve” category antibiotics. By 2023, the WHO aims for at least 60% of all antibiotic consumption to come from the Access group [13].

Cost analysis

The cost of individual antimicrobial agents (AMAs) used during the study was collected from the pharmacy. The average cost per patient and average cost per day analysis were done in the study. This study gathered only the cost of AMAs utilized and did not include any expenses related to hospitalization, investigations, procedures, or other costs.

Statistical analysis

Data entry was performed in a Microsoft Excel spreadsheet (Microsoft Corporation, Redmond, USA), followed by analysis conducted with IBM SPSS Statistics for Windows, Version 23 (Released 2020; IBM Corp., Armonk, New York, United States) [14]. The descriptive statistics were presented using frequencies and percentages.

## Results

A total of 417 participants took part in this study. There were 204 (49%) males and 213 (51%) females. Around 182 (44%) participants belonged to the 18-40 years age group. The socio-demographic profile of the study participants has been depicted in Table 1.

**Table 1:** Distribution of study participants according to socio-demographic and clinical variables

Variable		Frequency (n = 417)	Percentage
Gender	Male	204	49.00
	Female	213	51.00
Age group (in years)	18-40	176	43.00
	41-60	182	44.00
	>60	53	13.00
Educational Status	Illiterate	43	10.30
	Primary	122	02.92
	Secondary	101	24.22
	Graduate	151	36.21
Occupational Status	Salaried class	191	46.00
	Farmer	78	19.00
	Businessman	58	14.00
	Homemaker	27	06.00
	Others	63	15.00
Co-morbidities	Diabetes Mellitus	117	28.00
	Hypertension	56	13.00
	Dyslipidemia	28	07.00
	Hypothyroidism	25	06.00
	Gout	17	04.00
	No comorbidities	174	42.00
Body Mass Index (BMI) (kg/m^2^)	Normal	91	21.82
	Abnormal	326	78.18
Length of hospital stay (in weeks)	1	45	10.79
	1 – 2	188	45.08
	>2	184	44.12

**Figure 1:**
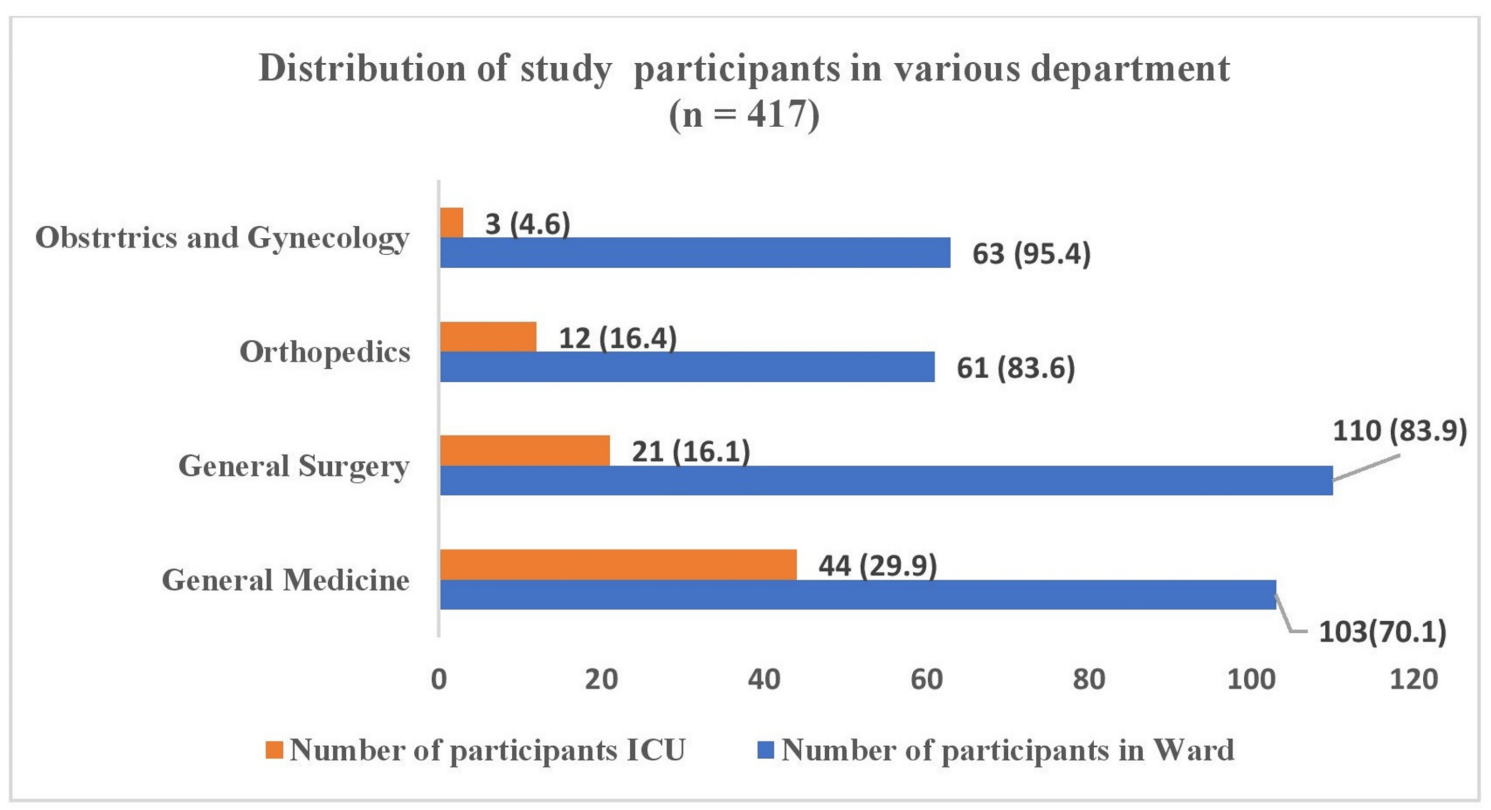
Distribution of study participants according to admission into various departments - n (%)

The participants were admitted under four clinical departments, namely General Medicine, Surgery, Orthopedics, and Obstetrics and Gynaecology (O&G) in both the ward and intensive care unit (ICU), as depicted in Figure 1.

In this study, a total of 1261 antimicrobials were prescribed among 417 patients, as outlined in Table 2. It was found that ceftriaxone was the most common antimicrobial used in Medicine 56 (18.5%) and Surgery 49 (18.2%). In orthopaedics and O&G, the most common antimicrobials used were cefuroxime 37 (20.3%) and cefixime 32 (22.2%), respectively. Piperacillin-tazobactam 59 (16.3%) was the most frequently utilized antimicrobials in the ICU (Table 2).

**Table 2:** Frequency of antimicrobials prescribed in study participants AMAs: antimicrobial agents

AMAs	Medicine (n=103)	Surgery (n=110)	Orthopedics (n=61)	Obs & Gyn (n=63)	ICU (n=80)
Acyclovir	6 (1.9%)	-	-	-	4 (1.1%)
Amikacin	12 (3.9%)	7 (1.5%)	27 (14.8%)	-	11 (3.0%)
Amoxicillin-Clavulanic acid	15 (4.9%)	35 (12.9%)	4 (2.2%)	-	-
Azithromycin	7 (2.3%)	-	-	-	2 (0.6%)
Cefixime	8 (2.6%)	16 (5.9%)	-	32 (22.2%)	-
Cefotaxime	8 (2.6%)	6 (2.2%)	-	27 (18.8%)	25 (6.9%)
Cefoperazone-Sulbactam	25 (8.3%)	18 (6.7%)	4 (2.2%)	-	30 (8.3%)
Ceftriaxone	56 (18.5%)	49 (18.2%)	9 (4.8%)	24 (16.7%)	21 (5.8%)
Ceftriaxone-Tazobactam	11 (3.6%)	9 (3.3%)	17 (9.3%)	2 (1.4%)	35 (9.7%)
Cefuroxime	14 (4.6%)	43 (15.9%)	37 (20.3%)	-	-
Clindamycin	7 (2.3%)	7 (2.6%)	-	10 (6.9%)	13 (3.6%)
Clotrimazole	-	-	-	11 (7.6%)	-
Colistin	-	-	-	-	12 (3.3%)
Clarithromycin	5 (1.7%)	-	-	-	-
Doxycycline	29 (9.6%)	-	-	-	32 (8.8%)
Gentamycin	-	4 (1.5%)	23 (12.6%)	-	3 (0.8%)
Itraconazole	-	-	-	-	6 (1.7%)
Linezolid	7 (2.3%)	14 (5.2%)	-	-	17 (4.7%)
Metronidazole	17 (5.6%)	28 (10.4%)	24 (13.2%)	22 (15.3%)	16 (4.4%)
Meropenem	16 (5.3%)	4 (1.5%)	-	-	33 (9.1%)
Mupirocin	-	-	-	16 (11.1%)	-
Nitrofurantoin	6 (1.9%)	-	4 (2.2%)	-	3 (0.8%)
Ofloxacin	16 (5.3%)	-	13 (7.1%)	-	7 (1.9%)
Piperacillin-Tazobactam	30 (9.9%)	28 (10.4%)	22 (12.1%)	-	59 (16.3%)
Rifaximin	8 (2.6%)	-	-	-	-
Teicoplanin	-	-	-	-	7 (1.9%)
Tigecycline	-	-	-	-	9 (2.5%)
Vancomycin	-	-	-	-	17 (4.7%)
Total drugs	303	268	184	144	362

In this study, it was found the overall total number of drugs prescribed per prescription was 2006 and the average number of drugs per prescription was 4.81 (Table 3).

**Table 3:** WHO Core Drug Prescribing Indicators *Since the study was on drug utilization of antimicrobials, the percentage of encounters with an antibiotic is 100%.

Prescribing indicators	WHO optimal values	Values in this study
Average number of drugs per prescription	1.6-1.8	4.81±0.90
Percentage of drugs prescribed by generic names	100%	12.3%
Percentage of encounter with an antibiotic	20.0-26.8%	100%^*^
Percentage of encounter with an injectable antibiotic	13.4-24.1%	55.3%
Percentage of drugs prescribed from essential drug list	100%	87.5%

From the WHO Access, Watch, and Reserve classification (AWaRe Index), the Watch group antibiotics were utilized 800 times (65%) followed by the Access group 359 (29%) and the Reserve Group 59 (5%). The same has been outlined in Table 4.

**Table 4:** Utilization pattern of antibiotics as per WHO Access, Watch, Reserve (AWaRe) groups In total, 1261 antimicrobials (1234 antibiotics, 15 antifungals, 10 antivirals) were used in this study. n=1234 (total no. of antibiotics used) 16 (1%) mupirocin ointments are not available in the WHO AWaRe Index.

Access	%	Watch	%	Reserve	%
1) Metronidazole	107 (8.7%)	1) Ceftriaxone	233 (18.8%)	1) Linezolid	38 (3%)
2) Doxycycline	61 (4.9%)	2) Piperacillin+tazobactam	139 (11.3%)	2) Colistin	12 (1%)
3) Amikacin	57 (4.6%)	3) Cefuroxime	94 (7.6%)	3) Tigecycline	9 (0.7%)
4) Amoxicillin+Clavulanic	54 (4.4%)	4) Cefoperazone	77 (6.2%)		
5) Clindamycin	37 (3.0%)	5) Cefotaxime	66 (5.3%)		
6) Gentamicin	30 (2.4%)	6) Cefixime	56 (4.5%)		
7) Nitrofurantoin	13 (1.0%)	7) Meropenem	53 (4.3%)		
		8) Ofloxacin	36 (2.9%)		
		9) Vancomycin	17 (1.4%)		
		10) Rifaximin	8 (0.6%)		
		11) Azithromycin	9 (0.7%)		
		12) Teicoplanin	7 (0.6%)		
		13) Clarithromycin	5 (0.4%)		
Total	359 (29%)		800 (65%)		59 (5%)

In this study, it was observed that diarrhoea 25 (46.3%) and rashes 11 (20.4%) were the most common ADRs, as assessed by the World Health Organization-Uppsala Monitoring Centre (WHO-UMC) Causality Assessment framework. The cephalosporin 31 (57%) group of drugs caused the maximum ADRs. All the ADRs observed were nonserious in this study, as outlined in Table 5.

**Table 5:** World Health Organization-Uppsala Monitoring Centre (WHO-UMC) Causality Assessment Scale among the study participants

Suspected drug	Adverse Drug Reaction (ADR)	Categories	Frequency
Ceftriaxone	Diarrhea	Possible	9
	Itching	Probable	6
	Vomiting	Possible	2
	Rash	Probable	4
Ceftriaxone+Tazobactam	Diarrhea	Possible	1
	Itching	Probable	1
Cefoperazone+Sulbactam	Diarrhea	Possible	1
Piperacillin+Tazobactam	Diarrhoea	Possible	3
	Rashes	Probable	6
	Itching	Probable	1
Cefuroxime	Diarrhea	Possible	7
Linezolid	Diarrhea	Possible	4
Meropenem	Constipation	Possible	6
	Itching	Probable	1
	Rashes	Possible	1
Ofloxacin	Fixed drug eruption	Probable	1
Total			54

In this study, it was found that the cost of antimicrobial treatment was highest among the patients admitted to ICU. The average cost per patient was 6450 (INR) while the cost per day according to length of stay in ICU was 430 (INR), as depicted in Table 6.

**Table 6:** Average cost of antimicrobial treatment for study participants in various departments INR: Indian Rupees

Department	Average cost per patient (INR)	Average cost per patient per day (INR)
Medicine (n=103)	2092 ±626.9	174.3±52.24
Orthopedics (n=61)	1722±516.2	107.2±30.3
Surgery (n=110)	1265±378.6	90.3±27.0
Obs & Gyn (n=63)	660±408.8	73.3±45.4
ICU (n=80)	6450±3877.7	430±258.5

## Discussion

In the recent studies by Ramachandra et al [15] and Khan et al [16], the majority of participants were above 40 years of age. The mean age of the study population at the baseline visit was 44.26 ± 14.82 years. Most of these individuals were 40-65 years of age. The age range of the study participants is consistent with the above-mentioned studies. It indicated that middle-aged individuals had an increased likelihood of hospital admission and antibiotic administration. The mean BMI of the study participants was 27.58 ± 3.76 kg/m2, with a range of 23.63-32.11 kg/m2. Most of the study participants were overweight and obese (78.18%). The present study findings also concord with Ramachandra et al's [15]. This finding indicates that individuals with a higher BMI are more susceptible to hospitalization and pharmacotherapy in contrast to those with a normal BMI. Gender distribution was almost equal in the study population (Table 1). Recent studies by Cangini et al [17], Bhardwaj et al [18], Solanki et al [19], and Kotwani et al [20] show that the risk of prolonged hospitalization increases with the advancing age of participants and increased BMI.

Just 80 participants required ICU admission. The overall average hospital stay was 13.16 ± 7.39 days with a range of 4-24 days, whereas the average length of stay in the ICU was around 15 ± 3 days, Orthopaedics 16 ± 4 days, Surgery 14 ± 3 days, Medicine 12 ± 3 days, and O&G 9 ± 2 days. The patients were assessed from the wards and ICUs of the Medicine, Surgery, Orthopedics, and Obstetrics and Gynecology departments of the institution. 103 participants were included from the Medicine ward, and the other three departmental wards provided 110, 61, and 63 patients, respectively. The number of participants requiring ICU admission was 44, 21, 12, and 3 from the Medicine, Surgery, Orthopedics, and O&G departments, respectively, making a total of 80 ICU admissions (Figure 1).

Third-generation cephalosporins were the most common antibiotic administered to in-patients. Beta-lactams 772 (61%) outnumbered all other antibiotics in the context of drug utilization in wards and ICUs (Table 02), which is similar to the findings of Simahdri et al [21], where beta-lactams (60.8%) were predominantly prescribed. The drugs were ceftriaxone (parenteral), cefuroxime (oral), and cefixime (oral). The most utilized antimicrobial in ICU patients was broad-spectrum penicillin, i.e., a fixed-dose combination (FDC) of piperacillin-tazobactam. Cangini et al [17] found that ceftriaxone and piperacillin were the most common antibiotics prescribed to most of their participants. Bhardwaj et al [18] revealed that the majority of their patients were treated with cefuroxime and ceftriaxone. Solanki et al [19] found that cefixime and ceftriaxone were the most commonly utilized drug. According to Kotwani et al [20], ceftriaxone, cefoperazone, and broad-spectrum antibiotics like doxycycline and tigecycline were rampantly administered compared to other antibiotics. Moss et al [22] suggested that penicillins were the most commonly prescribed antibiotic in the in-patient settings. Likewise, Rahman et al [23] found that ceftriaxone and piperacillin-tazobactam were the most common antibiotics to be used in ICU patients. This study's findings are similar to the findings of the above-mentioned studies.

In this study population, a total of 1261 antimicrobials were administered. Of them, the majority were utilized in the ICU (362), followed by the departments of medicine (303), surgery (268), orthopaedics (184), and obstetrics and gynaecology (144). The average number of drugs per prescription was 4.81 ± 0.90 (Table 03). As per Vineel et al [24], the average number of drugs prescribed was 8.2. Khan et al [16] found that on average 2.74 drugs were prescribed per encounter. In all the studies, including the present study, the average number of drugs per prescription is higher than the standard WHO optimal​​​​​​ value of 1.6-1.8. This may be attributed to the higher prevalence of comorbid conditions. An elevated result beyond the typical threshold is indicative of polypharmacy.

The WHO AWaRe classification was used to analyze the antibiotic prescribed in this study (Table 4). The results showed that the majority of the drugs used were from the Watch group (65%), which is a broad-spectrum group of antibiotics, followed by the Access group (29%), which is a narrow-spectrum group, and the least number of drugs were utilized from the Reserve group (5%). The use of antibiotics from the Watch group in this study was less compared to Ali et al (70%) [25] but higher than Negi et al (38.2%) [26]. The findings of antibiotic usage from the Access group in this study were higher than in Ali et al [25] - 21.4% and 17.6%, respectively - but lower than Negi et al [26], which revealed 57.6% of antibiotics utilized from the Access group. Antibiotic usage from the Reserve group in this study is almost similar to Negi et al (4.3%) [26] study but very low compared to Ali et al (11.6%) [25], which is a positive sign since as per the WHO Reserve group of antibiotics should be used only as last resort when other treatments and options have failed in highly specific circumstances. However, as per WHO recommendations, antibiotic usage from the Watch category should not be more than 40%, and usage from the Access group should be a minimum of 60%, which is inconsistent with this study's findings. It may be because the study was carried out in a tertiary care hospital in an in-patient setting, including the corresponding ICUs, where more complicated and critical cases with higher comorbid conditions are presented that need aggressive treatment as per physicians, and hence, have a greater chance of prescribing broad-spectrum antimicrobials.

In this study, the most common antibiotics used from the Access group were metronidazole, ceftriaxone, and piperacillin/tazobactam from the Watch group and colistin from the Reserve group (Table 4). The findings are consistent with Negi et al [26]. The present study's results and the analysis of results of other studies show that the availability and consumption of the Watch group of antibiotics in all types of health facilities in India are much higher than that of the Access group of antibiotics. Therefore, proper awareness of the AWaRre Index and its impact on antimicrobial utilization should be done at the institutional level at regular intervals for healthcare professionals along with effective antimicrobial stewardship measures.

In this study, 54 mild cases of ADRs were noted in the ADR form (Table 5). The causality assessment was done using the WHO-UMC assessment scale. The findings revealed that the majority of the cases of ADRs were of antibiotic-associated diarrhoea 25 (46%). The cephalosporin group of antibiotics caused the majority of the adverse effect of diarrhoea 31 (57%), which is consistent with Bergogne-Berezin et al [27]. Among cephalosporins, ceftriaxone caused nine (16%) of the diarrhoea cases, which is similar to Fredua-Agyeman et al [28], where the value was eight (17%). All cases were non-serious, and resolution occurred by withdrawing the specific antibiotic, additional management, or switching over to another antibiotic.

The average cost of antibiotics (Table 6) was highest in the ICU (6450 INR), followed by Medicine (2092 INR), Orthopaedics (1722 INR), Surgery (1265 INR), and Obstetrics and Gynaecology (660 INR) departments. Patra et al [29] found that in India the total average cost of antibiotics varies from 2425 INR to 7826 INR and the majority of the cost comes from the ICU due to the seriousness and complexity of cases, which requires an aggressive approach with multiple drugs. The present study's findings are similar.

This study had some strengths. First, it had a lower attrition rate. Only 26 participants were lost to follow-up. 94.1% of participants completed the study and were included in the final analysis. Second, the enrolled participants visited for the confirmation of their clinical symptoms. Data were collected and any ADR found was mentioned, which might have gained their trust and encouraged the participants to adhere to the study. Third, the study covered the in-patient departments and ICUs of the four busiest departments of the institution, i.e., Medicine, Surgery, Orthopedics, and Obstetrics and Gynecology. Hence, a wide variety of prescriptions were obtained to evaluate. The patients with Over the Counter (OTC) medications and incomplete information on bed head tickets were excluded, considering the high risk for bias. Fourth, the study compared and contrasted all four departments for their drug utilization patterns, cost of antibiotics used, WHO core drug prescribing indicators, duration of stay, average antibiotic cost per encounter per day, and incidence of ADRs.

This study had some limitations as well. First, the sample size was small, considering the tertiary care hospital and the study duration. Second, this study did not correlate the socio-demographic characteristics and clinical parameters with the cost of treatment and number of antibiotics used. Third, this study could not quantify the direct and indirect costs of treatment for all of the study participants. Fourth, it could not assess the quality of life for the study participants.

## Conclusions

the most common group of antimicrobials used were beta-lactams in this study. Ceftriaxone, cefuroxime, and piperacillin-tazobactam were the most common drugs to be prescribed. It was found that this study does not correspond with the optimal values of WHO core drug use prescribing indicators. None of the participants had any serious adverse effects related to antimicrobials. It is anticipated that this study will contribute to the development of a more effective antimicrobial stewardship programme. However, further studies with larger sample sizes and longer study durations with frequent intervals are warranted to validate and generalize the study results and provide useful updates to the in-house healthcare professionals and policymakers.
